# Reconceptualizing employee engagement: toward a relational, existential alternative

**DOI:** 10.3389/fpsyg.2026.1975249

**Published:** 2026-10-28

**Authors:** Pyounggu Baek

**Affiliations:** Kookmin University, Seoul, Republic of Korea

**Keywords:** Bourdieu, engagement, existential choice, habitus, jingle-jangle, Sartre

## Abstract

Employee engagement has become one of the most influential constructs in organizational scholarship, yet its centrality has rarely been matched by conceptual scrutiny. This article examines employee engagement through three converging issues: a largely unexamined presumption that engagement is inherently good, a definitional ambiguity produced by persistent jingle-jangle phenomena and by a growing gap between what engagement measures claim to capture and what they actually measure, and a boundaryless expansion sustained by an alliance with managerialist discourse. Building on this diagnosis, the article proposes a transformative reconceptualization that integrates Sartrean existential choice with Bourdieusian field-habitus theory, repositioning employee engagement from a manageable psychological state to an active, relational practice of existential self-definition within organizational fields. Three propositions specify how this reconceptualization redirects research design toward field position, capital, and habitus rather than measurable psychological states, and an illustrative application shows how apparently identical engagement scores can reflect differently structured existential and relational conditions. The article closes by situating this diagnostic pattern within the broader literature on dominant, ambiguous, and expansive organizational concepts, and by outlining the theoretical, methodological, and practical implications of treating engagement as a relational practice rather than a manageable psychological state.

## 1 Introduction

Owing to advances in digital technology, boredom is no longer a viable state for those of us living in the present. Despite an abundance of stimulation, sustaining attention has paradoxically become harder to achieve. Frequent task switching and constant interruptions from emails and messages deplete employees’ cognitive resources (Leroy, 2009). The contrast between shallow work and deep work (Newport, 2016) captures a broader paradox of contemporary workplaces: sustained, meaningful engagement has become difficult to achieve precisely when organizations need it most.

This paradox has long attracted the interest of organizational researchers, who have approached employee engagement chiefly through two theoretical traditions. Kahn (1990) identified meaningfulness, safety, and availability as the three psychological conditions that give rise to engagement, while the Job Demands–Resources (JD-R) model defines disengagement as an imbalance between job demands and resources (Demerouti et al., 2001; Bakker et al., 2023), later refined by Crawford et al.'s (2010) distinction between challenge and hindrance demands. In the more than three decades since Kahn's (1990) seminal contribution, engagement has come to influence both academic and practitioner communities across industrial-organizational psychology, organizational behavior, organization studies, human resource development, public administration, and education (Sambrook, 2021). Within the JD-R tradition in particular, where an input-process-output logic is widely accepted, engagement research appears to follow an orderly trajectory of cumulative theoretical development (Bakker et al., 2023).

Yet the more thoroughly a concept comes to dominate a field’s discourse, the more urgently it warrants critical scrutiny (Alvesson and Blom, 2022). A growing body of critical scholarship has already begun this scrutiny. Truss et al. (2013) observe that engagement research pursues both organizational performance and individual well-being without adequately theorizing the tension between the two. Others have pointed to the concept’s unitarist and managerialist underpinnings and its blurred conceptual boundaries (George, 2011; Keenoy, 2014). Shuck et al. (2016) go further, arguing that engagement discourse obscures questions of power, privilege, and inequality by leaving unexamined who possesses the enabling conditions for engagement and whose engagement is deemed valuable. Most recently, Wittenberg et al. (2024) show that mainstream engagement research remains constrained by positivist assumptions of objectivity, stability, and decontextualized universality—assumptions that structurally marginalize the experiences of diverse and underrepresented stakeholders.

These critiques, taken individually, illuminate important but disparate limitations. What has not yet been attempted is a systematic synthesis of the issues they raise, nor a constructive theoretical alternative that moves beyond diagnosis. This article takes up both tasks through three aims. First, it synthesizes these scattered critiques into a single diagnostic account of employee engagement. Second, it proposes a relational reconceptualization that renders the diagnosed problems tractable rather than merely naming them. Third, it derives from this reconceptualization a set of propositions that reorient research design and organizational practice. The significance of this undertaking is both theoretical and practical. Theoretically, it shifts the focus of engagement research away from refining the measurement of a state presumed to be good and toward asking how, under what structural conditions, and at what cost engagement is enacted. Practically, it invites organizations to reconsider interventions that treat engagement as a lever to be pulled and to attend instead to how recognition and capital are distributed within the organizational field. As Section 3.4 clarifies, these are not parallel resources: capital is what members invest, whereas recognition is the process through which the field values that investment.

Section 2 examines employee engagement scholarship through three interrelated issues: the field’s largely unquestioned presumption of engagement’s inherent goodness, the definitional ambiguity produced by persistent jingle-jangle phenomena and by a growing gap between what engagement measures purport to capture and what they actually measure, and the concept’s boundaryless expansion in alliance with managerialist discourse. Building on this diagnosis, Section 3 proposes a transformative reconceptualization of engagement that integrates Sartrean existential choice with Bourdieusian field-habitus theory, reframing engagement not as a manageable psychological state but as an active, relational practice of existential self-definition within organizational fields. Section 4 discusses the theoretical, methodological, and practical implications of this reconceptualization, together with its challenges, limitations, and directions for future research; Section 5 concludes by situating the diagnosis within the broader literature on dominant, ambiguous, and expansive organizational concepts.

## 2 Employee engagement under scrutiny: three converging issues

### 2.1 Issue 1: the presumption of inherent goodness

The prevailing appeal of employee engagement rests on a largely unexamined premise: that engagement is intrinsically desirable, and that more of it is simply good. This premise echoes the normative goodness long attributed to leadership (Blom and Alvesson, 2015) and resonates with a broader assumption in human resource development that development, in excess, can never cause harm (Rigg et al., 2007). The premise is especially compelling in contemporary workplaces, where sustained attention and meaningful involvement have become difficult to achieve; a concept promising to restore them is, almost by definition, attractive and difficult to resist. Just as defining leadership primarily by its positive outcomes creates a self-referential shield that forecloses critical scrutiny of leadership itself (Spoelstra and ten Bos, 2011; van Knippenberg and Sitkin, 2013), engagement’s presumed goodness has similarly discouraged its own critical examination—so much so that even engagement researchers have begun to ask why this scrutiny was so long delayed (Shuck et al., 2016).

This presumed goodness is reinforced, in turn, by the sheer proliferation of engagement measures. Representative definitions include Kahn’s (1990, p. 694) “harnessing of organization members’ selves to their work roles”, Schaufeli et al.’s (2002, p. 74) “positive, fulfilling, work-related state of mind that is characterized by vigor, dedication, and absorption”, and Shuck and Wollard’s (2010, p. 103) “cognitive, emotional, and behavioral state directed toward desired organizational outcomes”; yet even within the conceptual literature, a precise definition often proves elusive (Lee et al., 2017). Rather than resolving this ambiguity, researchers have instead invested in developing and validating instruments, reflecting engagement research’s growing dependence on operational rather than conceptual definition (Kerlinger and Lee, 2000). This pattern reflects the resonance of engagement within a positivist research culture increasingly oriented toward career generation rather than knowledge generation (Suddaby, 2010; Alvesson et al., 2017).

The resulting instrument landscape (Table 1) reveals several patterns of dominance. UWES-9—originally a measure of work engagement (vigor, dedication, absorption) but frequently reported as employee engagement—remains the most widely used instrument (Schaufeli et al., 2006), alongside variants such as the Shirom-Melamed Vigor Measure (Shirom, 2003). Saks (2006) proposed a two-dimensional model distinguishing job from organizational engagement, while later instruments have extended the construct to include physical (Rich et al., 2010) and social (Soane et al., 2012) dimensions. Compared with other pervasively diffused organizational concepts examined in prior critical concept research—such as leadership, strategy, and institution (Alvesson and Blom, 2022)—engagement has not fully displaced satisfaction, involvement, or commitment (cf. Yang et al., 2021, on social impact in accounting). Its dominance is therefore not one of replacement but of discourse: engagement functions as a superordinate construct that subsumes adjacent concepts (Christian et al., 2011), a pattern of construct proliferation in which measurement tools multiply even where conceptual territory is already well occupied (Shaffer et al., 2016).

**Table 1:** Construct dimensions of engagement measurement tools.

Instrument	Source	Origin	Dimensions	Explanatory note
UWES (Utrecht Work Engagement Scale)-9	Schaufeli et al. (2006)	Academic	Vigor, dedication, absorption	Measures work engagement rather than employee engagement, yet remains the most frequently used instrument in employee engagement research.
SMVM (Shirom-Melamed Vigor Measure)	Shirom (2003)	Academic	Physical strength, emotional energy, cognitive liveliness	Measures vigor as a stand-alone construct and is used as a variant alongside the UWES.
Job and organization engagement scales	Saks (2006)	Academic	Job engagement, organization engagement	Distinguishes job engagement from organization engagement as two role-specific forms.
Employee Engagement Scale	Shuck et al. (2017a)	Academic	Cognitive, emotional, behavioral	Operationalizes the tripartite definition of Shuck and Wollard (2010).
Psychological engagement scale	May et al. (2004)	Academic	Cognitive, emotional, physical	Operationalizes the personal engagement of Kahn (1990).
Job Engagement Scale	Rich et al. (2010)	Academic	Cognitive, emotional, physical	Extends the three forms of investment in Kahn (1990) to the prediction of job performance.
ISA Engagement Scale	Soane et al. (2012)	Academic	Intellectual, social, affective	Adds a social dimension to the cognitive-affective core.
Gallup Q12	Harter et al. (2002)	Practitioner	Twelve items on perceived workplace conditions	Overlaps substantially with overall job satisfaction and is analyzed at the business-unit level.
Say–Stay–Strive model	Aon Hewitt (2015)	Practitioner	Say, Stay, Strive	Combines commitment, intention to remain, and discretionary effort in a six-item index.

The two most widely adopted practitioner frameworks intensify rather than mitigate this pattern. The Gallup Q12 comprises 12 items on perceived workplace conditions and has been examined at the business-unit level; its developers label the construct “satisfaction-engagement” and report that the items account for a substantial proportion of the variance in overall job satisfaction (Harter et al., 2002, p. 268). The jingle problem described under Issue 2 is thus evident in the very name of the instrument. The Aon Hewitt model defines engagement through three observable facets—Say, Stay, and Strive—operationalizes them as a six-item index with two items per facet, and pairs this index with six clusters of drivers (Aon Hewitt, 2015). Its developers describe engagement as a combination of constructs long studied under other names, including affective and continuance commitment, motivation, and organizational citizenship behavior. Both frameworks thus exemplify the superordinate-construct pattern noted above, and both position engagement as an organizational outcome to be diagnosed and improved—the managerial orientation examined under Issue 3. Viewed through the lens proposed below, what they capture is merely the distribution of responses, not the choices and positions that produce those responses.

### 2.2 Issue 2: definitional ambiguity

Employee engagement does not suffer from a shortage of definitions but from their excess. Two distinct forms of ambiguity compound this problem: jingle-jangle confusion, and a growing gap between what engagement measures claim to capture and what they, in practice, measure.

The jingle problem is evident in Bailey et al.’s (2017) systematic review of 214 engagement studies, which identified the UWES—a measure of work engagement—as the most widely adopted measure, used in 148 of the studies reviewed and often reported under the broader label of employee engagement. This same term is applied inconsistently to conceptually distinct relationships: engagement with work, satisfaction with a job role, and commitment to an organization are frequently treated as interchangeable despite tracking different psychological referents (Shuck et al., 2013). The jangle problem runs in the opposite direction: work engagement, personal engagement (Kahn, 1990), and general employee engagement are often given different names despite converging on a similar underlying phenomenon—the investment of cognitive, emotional, behavioral, and physical energy in one’s work (Rich et al., 2010). This naming inconsistency both signals theoretical redundancy, famously described as old wine in new bottles (Macey and Schneider, 2008), and obscures the proposition that engagement may not differ meaningfully from general job attitudes already well studied under other names (Newman et al., 2011).

A second, related ambiguity concerns the gap between what engagement measures are presumed to capture and how that capture is subsequently used. Consider a hypothetical study in which work engagement (vigor, dedication, absorption), measured via the UWES, is found to be positively associated with both transformational leadership and self-reported performance. The measurement itself targets a variable, temporary state of personal resource investment; yet the study’s practical implications typically prescribe stable organizational interventions—specific leadership behaviors, new HR programs—that treat this fluid state as if it were a manageable organizational lever. This decoupling between what is measured (a momentary state, whether framed as psychological or behavioral) and what is prescribed (durable structural intervention) risks treating organizational members as adjustable mechanisms rather than as people whose energy fluctuates for reasons the instrument does not capture.

This decoupling connects to a further psychometric ambiguity: engagement sits uncomfortably between state and trait, and between attitude and behavior (Macey and Schneider, 2008; Newman and Harrison, 2008). Schaufeli’s conceptualization of engagement as a relatively persistent but not fixed state (Schaufeli et al., 2002) exemplifies this positioning, complicating the interpretation of test–retest reliability. Likewise, a hypothetical survey item such as “I am absorbed in my work” leaves unclear whether it states an attitude or a behavior (Macey and Schneider, 2008; Shuck et al., 2017b), a problem that extends beyond conceptual definition to raise concerns about measurement validity (Saks and Gruman, 2014). Ambiguity, in other words, is not a peripheral feature of engagement research but a structural one, embedded in the very measurement architecture the field relies on. One constructive response to this ambiguity, and arguably the most defensible within the prevailing paradigm, is contextualization: specifying the definition of engagement according to the purpose and setting of a given study, and ensuring that the dimensions measured are aligned with that definition. This principle is endorsed here. Contextualization, however, addresses ambiguity at the level of the individual study rather than at the level of the field. As long as studies routinely report work engagement under the label of employee engagement (Bailey et al., 2017), misalignment between definition and measured dimensions persists across the literature even where individual studies are internally coherent. The problem diagnosed here is therefore not only that definitions are ambiguous, but that definitions and dimensions are frequently decoupled.

### 2.3 Issue 3: boundaryless expansion and managerialist alliance

The third issue concerns scope. Engagement’s appeal is not confined to the workplace: comparable constructs have proliferated across school engagement (Fredricks et al., 2004), student engagement (Kahu, 2013), teacher engagement (Klassen et al., 2013), customer engagement (Brodie et al., 2011), and civic engagement (Adler and Goggin, 2005), such that engagement can plausibly be described as covering everything, and therefore nothing in particular (Learmonth and Morrell, 2019). Engagement in academic settings is not equivalent to engagement in office work or in other industries, and each domain arguably warrants a definition and set of dimensions suited to its own context. The difficulty arises when such context-specific constructs are subsumed under a single, unmarked label and findings from one domain are generalized to another without specifying the boundary conditions of either. Accordingly, the present analysis is confined to employee engagement in work organizations; its diagnosis and reconceptualization are not intended to extend to student, customer, or civic engagement without further contextual specification. The boundaryless expansion of engagement itself rests on an implicit relational assumption: much as leadership presupposes a schema of leaders and followers, engagement discourse presupposes asymmetric relations—between teacher and student, company and customer, organization and employee (Alvesson and Spicer, 2016). The problem is not that such asymmetries exist, but that engagement discourse treats them as static, obscuring how actors’ positions within these relations shift across contexts and over time.

This assumption of stable, manageable relations underwrites what might be called a myth of manageability: a widespread confidence that engagement, once diagnosed, can be engineered through intervention. Yet this confidence tends to identify “the organization” as the primary beneficiary of engagement management, a framing that in practice privileges those already in dominant organizational positions (Siehl and Martin, 1984; Bartunek and Rynes, 2010) and reinforces the argument that engagement discourse insufficiently attends to power and privilege (Shuck et al., 2016).

The costs of this managerial framing become clearest when engagement is contrasted with a related but distinct construct: eudaimonic experience. Aristotle’s eudaimonia describes an activity pursued for its own sake, not a psychological state to be induced or managed. Csikszentmihalyi's (1975, 1990) flow—an experience of complete absorption independent of conscious intention—captures something closer to this eudaimonic quality than most engagement measures do; yet Kahn's (1990) engagement, and the instruments built on it, remain oriented primarily toward cognitive investment amenable to measurement and management (May et al., 2004). Treating a genuinely eudaimonic, individual experience as an organizationally manageable variable substitutes a narrower, instrumentalized construct for a richer one—and in doing so allies the concept of engagement with what has been described as the self-fulfilling myth of managerialism (Alvesson and Willmott, 2012; Grey, 2017), in which further layers of management are continually proposed as the solution to problems that management itself may have helped produce (Kantola and Seeck, 2011; Cunliffe, 2014).

Read together, these three issues are not independent. The presumption of inherent goodness forecloses scrutiny of the very ambiguity that Issue 2 documents, since a concept already accepted as good invites less definitional interrogation. That same ambiguity, in turn, licenses the boundaryless expansion described in Issue 3: a concept whose meaning shifts easily across contexts can be extended into new domains with little resistance. And this expansion, by generating ever more applications, publications, and measurement tools, further consolidates the concept’s presumed goodness and discursive dominance—closing the circle back to Issue 1. This mutually reinforcing pattern, in which unquestioned value, definitional slippage, and unchecked expansion sustain one another, is not unique to employee engagement; it recurs across a recognizable class of concepts in organizational scholarship, a possibility this article returns to in Section 5.

## 3 Toward a relational reconceptualization: Sartrean-Bourdieusian engagement

### 3.1 Why Sartre, why Bourdieu

The three issues diagnosed above are not merely definitional or measurement problems; they are symptomatic of two deeper epistemological commitments that dominant engagement research rarely examines. The first concerns how the field conceives of the human psyche. Since Aristotle first distinguished the soul—as the form of the body—from a higher-order, disembodied mind (Russell, 1979), organizational research has largely inherited a picture of the person as decomposable into discrete faculties—cognition, emotion, behavior—that can be isolated, measured, and summed. This picture presupposes a notion of normality that is rarely made explicit: organizational research, including engagement research, has disproportionately sampled white-collar, professional employees (Kessler and Gutworth, 2023), rendering blue-collar experience comparatively invisible, and has treated Western samples as an unmarked default against which non-Western contexts require explicit specification (Schimmelpfennig et al., 2025). An alternative is available in traditions that resist strict mind–body dualism; Zhu Xi’s account of the mind as integrating essential nature and functional emotion, for instance, dissolves the state–trait and attitude-behavior splits documented in Issue 2 without requiring that the person first be decomposed into separable components (Virág, 2019; Angle and Tiwald, 2020). Existentialist philosophy offers a comparable, and for organizational scholarship more familiar, alternative: rather than asking what discrete faculties compose a person, it asks how a person constitutes themselves through choice.

The second commitment concerns how the field conceives of organizations. The still-dominant definition of organizations as goal-directed social entities deliberately structured and coordinated (Daft, 2021) reflects a modernist perspective committed to linear epistemology: universal principles, closed systems whose whole equals the sum of their parts, and futures predictable through stable causal relationships (Jayanti, 2011). This perspective underwrites the input-process-output logic that, as noted in the Introduction, makes engagement research feel orderly and cumulative even as its foundational ambiguities go unresolved (Bakker et al., 2023). It also marginalizes holism—attentiveness to interaction among system components, context, history, and human agency—that a purely linear model cannot accommodate (Iles and Yolles, 2003; Jayanti, 2011; Yawson, 2013).

These two epistemological commitments are not incidental to the mutually reinforcing pattern described at the end of the previous section; they are its condition of possibility. A psychology that treats persons as bundles of measurable states makes engagement’s definitional excess tractable—indeed profitable—to instrument-builders. A modernist organizational epistemology that assumes manageable, linear causation makes engagement’s boundaryless expansion appear as a solvable engineering problem rather than a category error. Addressing employee engagement’s conceptual difficulties therefore requires more than refining existing measures; it requires displacing the epistemological commitments that make those difficulties reproducible. This article proposes that Sartrean existentialism and Bourdieusian field-habitus theory, brought into dialogue, offer such a displacement: the former unsettles the decomposable, manageable self; the latter unsettles the linear, closed organization.

### 3.2 Existential choice: engagement as self-definition

Sartrean engagement, as used here, does not refer to *littérature engagée*, the tradition of politically committed French intellectual writing, but to the core concepts developed in *Being and Nothingness* (Sartre, 2018): existential choice, self-definition, and freedom in situation. On this view, human beings do not possess a fixed essence that determines how they will act; rather, existence precedes essence, and people constitute who they are through the choices they continually make (Sartre, 2007). Applied to engagement, this reframing has an immediate consequence: engagement is not a state that happens to an employee, induced by the right leader behavior or HR program, but an existential act by which employees give meaning to their work and, in doing so, define themselves.

This reframing dissolves rather than resolves the attitude-behavior and state–trait ambiguities documented in Issue 2. If engagement is neither a manageable attitude nor a discrete behavior but an ongoing existential stance, then asking whether a given instance of engagement is really an attitude or a behavior becomes the wrong question; attitude and behavior are, on this view, inseparable expressions of the same underlying choice. Nor does it matter, for this purpose, whether the object of that choice is a task (work engagement), a formal role (job engagement), or the organization as a whole (organizational engagement); all are occasions for the same existential act. What changes is not the taxonomy of engagement but the analytic priority: rather than modeling engagement as an outcome of antecedent variables, this reframing asks how and why employees choose to engage agentically, treating that choice—drawing on the concept of freedom in situation (Sartre, 2018)—as constitutive of employee identity within the organizational context.

### 3.3 Habitus: engagement as structured practice

Existential choice, however, is never made in a vacuum, a limitation Sartre’s critics, including Bourdieu himself, have long pressed (Bourdieu, 1990). Habitus supplies the missing structural half of this account. In Bourdieu’s framework, habitus is simultaneously a structured structure—the sedimented product of the agent’s history within the field—and a structuring structure—the dispositional lens through which members perceive and act within that field (Emirbayer and Johnson, 2008). Employees choose whether and how to engage, but the scope of that choice is shaped by their position within the organizational field, by the economic, cultural, social, and symbolic capital they hold or lack (Bourdieu, 1986, 1989; Emirbayer and Johnson, 2008), and by a habitus formed through repeated organizational experience without being fully fixed by it.

Habitus also offers a principled alternative to the state–trait debate documented in Issue 2: rather than asking whether engagement is momentary or enduring, habitus theory treats engagement as structured by repetition yet always open to revision through new practice, positioning it between state and trait by design rather than by measurement ambiguity. Read together with existential choice, this yields a relational conceptualization of engagement: neither a purely internal psychological state—the dominant view critiqued in Issue 1—nor a purely external structural determination, but a practice constituted at the intersection of individual choice and field position.

### 3.4 Working definition and propositions

On the basis of this integration, this article defines employee engagement as an ongoing, situated practice through which organizational members choose to invest themselves in their work within the possibilities and constraints set by their field position, capital, and habitus, and thereby define who they are within the organization. This definition differs from existing definitions in three respects. First, engagement is a practice rather than a state; it is therefore not something that employees possess at a given level. This does not deny that engagement has psychological and behavioral aspects. Existing definitions already encompass both: Shuck and Wollard (2010) include a behavioral component, and Macey and Schneider (2008) distinguish state from behavioral engagement. Yet such definitions typically either fold behavior into a “state” or treat it as a separable dimension to be measured alongside cognition and emotion. A practice, by contrast, comprises both how members experience their work and what they do in it; the psychological and the behavioral are therefore not two dimensions of engagement but inseparable aspects of a single situated practice (see Section 3.2). Second, engagement is relational: both its scope and its meaning depend on members’ positions in relation to others within the field. Recognition is central to this relationality: whereas capital is what members invest, recognition is the relational process through which the field confers value on, or withholds value from, that investment—a process Bourdieu (1989) analyzes as the conversion of capital into symbolic capital. Third, engagement is self-constitutive rather than merely instrumental: its value lies not only in what it delivers to the organization but also in what it makes of the person. Consistent with the principle that a definition should be aligned with the dimensions through which it is examined, this definition specifies its own analytic components: the choice to engage (addressed in P1), the field position and capital that bound that choice (P2), and the habitus and recognition through which it is sustained or eroded over time (P3). These components—rather than the cognitive, emotional, and behavioral dimensions summed in existing instruments—constitute the dimensions along which engagement as practice is to be examined. The following three propositions specify how this reconceptualization reorients engagement research.

*P1*. Employee engagement is not a psychological state to be measured but an existential act of self-definition; research questions should therefore ask how and why employees choose to engage, not merely whether or how much they are engaged.

*P2*. The scope of employees’ existential choice to engage is structured by their position within the organizational field and their portfolio of economic, cultural, social, and symbolic capital; engagement research should treat capital and field position as focal variables rather than as background controls.

*P3*. The existential choice to engage is sustained or eroded over time through the interplay between a habitus that is structured by repetition yet revisable and the recognition that the field allocates according to position; engagement research should therefore study engagement as a positional trajectory, in which choice, capital, and field position change together, rather than as a level observed repeatedly.

Two premises underlie P3. First, habitus is structured through repetition but is not fixed, so that engagement remains open to revision through new practice (Section 3.3). Second, recognition, the process through which the field confers value on what members invest, is allocated according to field position (Section 3.4). Designs that trace how engagement is enacted, recognized, and reinterpreted as positions shift are therefore preferable on theoretical as well as methodological grounds.

P3 does not, however, merely restate the familiar call for longitudinal designs. Repeated-measures and daily diary studies, motivated in part by the gain spirals of Conservation of Resources (COR) theory (Xanthopoulou et al., 2009; Hobfoll et al., 2018), already trace how a state fluctuates and how resources and engagement reinforce one another within persons. Yet what these designs follow over time is still a level, and what they model is reciprocal influence among relatively generic resources.

The present lens changes both the object and the explanation of temporal inquiry. First, the unit of analysis is not the score at each occasion but the trajectory of a person’s position and capital, since an identical score may be enacted from a rising, a stable, or an eroding position. Second, whether a gain spiral develops depends on whether the field recognizes what the employee invests. COR theory itself holds that those with fewer resources are less able to achieve gain (Hobfoll et al., 2018); the Bourdieusian lens adds that what counts as a resource is set by the field’s recognition, so that spirals are stratified by position rather than uniformly available, and the Sartrean lens adds that the meaning of a trajectory is continually reinterpreted through new choices. Third, habitus may persist beyond the conditions that formed it (Bourdieu, 1984), so that after a field is reconfigured, engagement can diverge from current conditions in ways that wave-based or daily designs would misread as noise. Longitudinal designs are therefore necessary but insufficient; they must be practice-based, tracing meaning, recognition, and position together.

### 3.5 Illustrative application

Consider two early-career employees, Employee A and Employee B, in the same firm, occupying similar formal roles and, on a UWES-based survey, reporting comparable levels of vigor, dedication, and absorption. This illustration is hypothetical and draws on no empirical data. A conventional analysis would treat this convergence as evidence that both employees are equally engaged and would look to shared antecedents—task design, workload, supervisor support—to explain the shared score. A Sartrean-Bourdieusian analysis asks a different question. Employee A, whose professional formation aligns closely with the firm’s dominant recruitment pipeline, occupies a field position consonant with the organization’s dominant habitus; the demanding tasks A reports as absorbing are also tasks the firm’s reward structure visibly recognizes, so A’s existential choice to engage carries relatively low cost and is continuously reinforced by accumulating symbolic capital. Employee B, whose prior professional habitus was formed under different institutional norms, occupies a comparatively marginal field position; the same tasks may absorb B’s attention just as fully, yet B’s choice to invest in them is not met with equivalent recognition, and B must additionally manage a habitus-field misalignment that the survey instrument does not register. Both employees may report identical UWES scores, but the existential and structural conditions of that engagement, and its sustainability, differ substantially. Where a conventional model would treat this difference as measurement noise or an omitted moderator, this reconceptualization treats it as the primary object of inquiry: not how much engagement exists, but through what choices, under what structural conditions, and at what cost to whom it is sustained.

## 4 Discussion

This section first sets out the theoretical, methodological, and practical implications of the reconceptualization, then reflects on its challenges and limitations, and finally outlines directions for future research.

### 4.1 Theoretical implications: beyond psychological reductionism

This reconceptualization creates a conceptual space in which two philosophical traditions frequently read as opposed—Sartrean existentialism and Bourdieusian structuralism—are brought into productive dialogue within a single account of engagement. In doing so, it repositions engagement from an individual psychological construct to a relational, sociological phenomenon without abandoning individual agency altogether. Habitus, as both structured and structuring, offers a principled route past the agency-structure dualism and, more specifically, past the micro–macro divide between social-psychological and sociological accounts of organizational life (Emirbayer and Johnson, 2008). Engagement research, on this view, becomes a candidate site for relational social science that captures individual agency and social structure simultaneously, rather than trading one off against the other.

### 4.2 Methodological implications: from psychological attribute to relational variable

Reconceptualizing engagement as relational requires a corresponding shift in research design. Rather than asking what this employee’s engagement level is, studies should ask what position an employee occupies within the organizational field, what capital they hold, and how closely their habitus aligns with the field’s dominant rules. This shift also implies the positional-trajectory designs specified in P3. Following Bourdieu’s distinction between primary and specific habitus (Emirbayer and Johnson, 2008), habitus is formed prior to organizational entry and subsequently reshaped within it, so such designs must capture both. Relevant questions include how a primary habitus formed before organizational entry clashes or aligns with an organization-specific habitus, and how engagement evolves as fields are reconfigured through restructuring or mergers in ways that alter members’ positions.

### 4.3 Practical implications: redesigning HR architectures for capital accessibility and habitus alignment

If engagement is an existential choice structured by field position, it cannot be produced directly through managerial intervention, yet the conditions under which that choice is made remain organizationally designable. This does not imply managerial abdication. It relocates organizational responsibility from engineering employees’ engagement to redesigning the HR architectures (selection, development, recognition, promotion, and performance-management systems) that shape who can afford to engage and at what cost. The role of organizations and HR functions therefore shifts from managing engagement to facilitating it, by enhancing the accessibility of capital, fostering habitus alignment, and easing, rather than engineering, the structural constraints on employees’ existential choices. Alignment is understood here as bidirectional: the field’s rules are themselves open to revision, so that alignment does not reduce to bringing employees’ dispositions into line with a dominant habitus. Because capital is typically distributed unevenly across an organizational field (Bourdieu, 1986, 1989), this facilitating role has three concrete components: policies that make economic, cultural, and social capital more visible and accessible, for example through structured learning and mentoring opportunities aimed at groups whose capital accumulation has historically been constrained; organizational cultures capable of accommodating diverse habitus, rather than treating one habitus—for example, that of graduates from prestigious universities—as an unmarked norm against which others are measured as deficient; and the redesign and, where appropriate, the discontinuation of surveillance and control systems that treat engagement as a target to be produced rather than a choice to be enabled.

Table 2 contrasts how interventions are designed under the prevailing state-based view and under the practice-based view proposed here.

**Table 2:** Intervention design under state-based and practice-based views of engagement.

Dimension	State-based view	Practice-based view
Object of intervention	Employees’ engagement level	Conditions under which choosing to engage is possible and sustainable
Diagnostic question	How engaged are employees?	Who can afford to engage, through what choices, and at what cost?
Typical evidence	Survey scores and benchmarking	Mapping of field position and capital; longitudinal narrative and ethnographic inquiry
Success criterion	Higher scores and performance gains	Broader and more equitable access to recognition and capital; sustainable engagement
Role of HR	Manager of engagement	Facilitator of enabling conditions

This practical reorientation stands in direct tension with the instrumental logic—engagement enhancement → performance gains → organizational profit—that currently organizes most workplace engagement initiatives. On the practice-based view, engagement is instead recognized as carrying eudaimonic value in its own right (Ryan and Deci, 2001), rather than functioning primarily as a lever for organizational outcomes. The practice-based view, in this sense, converts the concern for power and privilege raised by critical engagement scholars (Shuck et al., 2016) from an unresolved critique into an actionable design principle for organizational practice.

### 4.4 Challenges and limitations of the reconceptualization

Reconceptualizing engagement through the lenses of Sartre and Bourdieu entails challenges that warrant careful reflection. The first is theoretical. Bourdieu criticized philosophies of the subject for overlooking the social conditions of choice (Bourdieu, 1990, 2005), yet Sartre’s freedom in situation is not reducible to conditioning. The integration proposed here reconciles the two chiefly by attributing choice to the subject and its scope to the field; whether this division of labor is coherent, or whether it merely juxtaposes two accounts with conflicting ontologies, remains an open question. Critics may argue that habitus, as a system of durable dispositions, leaves little room for the radical freedom that Sartre affirms.

The second challenge concerns operationalization. Capital and field position are relational and context-dependent, and they therefore resist the standardized, portable measurement that made existing instruments attractive. Delimiting the boundaries of an organizational field and identifying its forms of capital require interpretive judgment, and different analysts may draw these boundaries differently. Moreover, the correspondence analysis proposed in Section 4.5 (Bourdieu, 1984) is by no means undemanding in terms of either data or analytical expertise.

The third challenge concerns practical uptake. Asking organizations to facilitate rather than manage engagement, and to make the distribution of capital visible, asks them to expose the power relations from which managerial authority is partly derived. Such proposals may be resisted or, ironically, absorbed as a new managerial program in which facilitation itself becomes a lever, the very outcome this article’s critique warns against.

Beyond these challenges, four further limitations bear directly on how these contributions should be read. First, this is a conceptual paper: Propositions 1–3 specify a research agenda but do not themselves constitute empirical evidence, and their value depends on subsequent qualitative and longitudinal work capable of testing them. Second, the theoretical apparatus proposed here draws on two European intellectual traditions, Sartrean existentialism and Bourdieusian sociology; although Section 3.1 gestures toward non-Western alternatives to mind–body dualism, the article’s central framework remains Western in origin, a tension worth acknowledging given its own critique of unmarked Western sampling defaults. Third, as a single-authored conceptual analysis, this article reflects one interpretive standpoint; the correspondence drawn in Section 5 between the three diagnosed issues and a broader class of organizational concepts is itself an interpretation open to contestation. Likewise, the Sartrean-Bourdieusian integration is offered as one possible response rather than the only one; alternative critical frameworks, such as Foucauldian power analysis or critical realism, might address the same three issues differently. Fourth, the working definition proposed in Section 3.4 is itself bounded. It is formulated for employee engagement in work organizations, and its components—choice, field position, capital, habitus, and recognition—are specified for that context. Engagement in educational, consumer, or civic settings, or in occupational fields whose forms of capital differ markedly from those considered here, would require context-specific elaboration of both the definition and the dimensions through which it is examined; no single definition, including the one proposed here, can capture every dimension of engagement across contexts.

### 4.5 Future research agendas

Two agendas follow directly. First, the designs proposed in P3 call for longitudinal narrative inquiry and ethnographic observation, which can be complemented by diary designs that additionally record recognition and changes in position and capital. Second, engagement research should resist further internal subdivision along measurement lines—an intellectual inbreeding that risks mistaking definitional proliferation for theoretical progress—and instead revive cross-disciplinary exchange, for instance by examining how digital technologies mediate the field positions and capital available to organizational members (Leroy, 2009; Bakker et al., 2023). Measurement itself warrants reconsideration: rather than summing Likert-scale items, techniques such as Bourdieu’s correspondence analysis, which maps the space of field positions onto the space of member dispositions (Bourdieu, 1984), may better capture habitus-field alignment than existing instruments.

## 5 Conclusion

This article has undertaken two related tasks. First, it examined employee engagement scholarship through three converging issues: a largely unexamined presumption of inherent goodness, a definitional ambiguity compounded by jingle-jangle confusion and by a decoupling between what engagement measures claim to capture and what they measure, and a boundaryless expansion sustained by an alliance with managerialist discourse. Second, building on this diagnosis, it proposed a transformative reconceptualization that integrates Sartrean existential choice with Bourdieusian field-habitus theory, repositioning engagement from a manageable psychological state to an active, relational practice of existential self-definition within organizational fields. It further specified a working definition of engagement as situated practice and derived three propositions, together with their implications for research design and organizational practice.

Without imposing a predetermined framework, this analysis finds that the three issues diagnosed above converge on a recognizable pattern in critical management studies. Alvesson and Blom (2022) describe a class of concepts they term hembig—organizational constructs that are simultaneously hegemonic, ambiguous, and big—citing leadership, strategy, and institution as examples. The correspondence is close: the presumption of inherent goodness (Issue 1) parallels hembig’s hegemonic status; the definitional excess documented in Issue 2 parallels its ambiguity; and the boundaryless, managerialist expansion described in Issue 3 parallels its bigness. Stated directly, this correspondence suggests that employee engagement meets the criteria for a hembig concept in Alvesson and Blom’s specific sense and does not merely share superficial features with one. Prior critical scholarship (Truss et al., 2013; Shuck et al., 2016; Wittenberg et al., 2024) has identified pieces of this pattern independently; what a hembig reading adds is a structural account of why these pieces recur together—hegemony concealing ambiguity, ambiguity licensing expansion, and expansion consolidating hegemony—a self-sustaining circularity rather than three unrelated flaws.

A paper that ranges across existentialist philosophy, field theory, and critical concept studies risks appearing expansive in exactly the manner it criticizes. This risk is worth naming rather than concealing. The reconceptualization proposed here is offered as a bounded theoretical proposal open to revision and refutation, not as a totalizing replacement for existing engagement research; its value should be judged by whether it generates tractable research questions, such as those specified in Propositions 1–3, rather than by the breadth of the theoretical territory it covers.

More broadly, this article cautions against the assumption that methodological sophistication guarantees theoretical rigor. Sartori (1970) distinguished the unconscious thinker, who adopts dominant tools uncritically, from the overconscious thinker, who imitates the rigor of natural science while overlooking the complexity of social phenomena; engagement research, like much of organizational scholarship, risks falling into either pattern when statistical technique substitutes for methodological reflection. Reframing employee engagement through the hembig lens is not intended as a rejection of empirical engagement research, nor as a defense of any single alternative construct such as Csikszentmihalyi’s flow, but as a form of productive provocation (Alvesson, 2013): an invitation to reexamine assumptions that have become taken for granted precisely because they were never seriously contested.

Employee engagement research need not abandon its accumulated empirical insights to take these challenges and limitations seriously. What it requires, this article has argued, is a willingness to treat engagement’s dominance, ambiguity, and reach not as signs of a maturing field but as questions the field has yet to ask of itself.

## References

[ref1] Adler R. P. Goggin J. (2005). What do we mean by "civic engagement"? J. Transform. Educ. 3, 236–253. doi: 10.1177/1541344605276792

[ref2] Alvesson M. (2013). The Triumph of Emptiness: Consumption, Higher Education, and Work Organization. Oxford: Oxford University Press.

[ref3] Alvesson M. Blom M. (2022). The hegemonic ambiguity of big concepts in organization studies. Hum. Relat. 75, 58–86. doi: 10.1177/0018726720986847

[ref4] Alvesson M. Gabriel Y. Paulsen R. (2017). Return to Meaning: A Social Science with Something to Say. Oxford: Oxford University Press.

[ref5] Alvesson M. Spicer A. (2016). The Stupidity Paradox: The Power and Pitfalls of Functional Stupidity at Work. London: Profile Books.

[ref6] Alvesson M. Willmott H. (2012). Making Sense of Management: A Critical Introduction. 2nd Edn London: Sage.

[ref7] Angle S. C. Tiwald J. (2020). “Moral psychology: Heartmind (*Xin*), nature (*Xing*), and emotions (*Qing*),” in Dao Companion to ZHU Xi's Philosophy, eds. Ng K. Huang Y. (Cham: Springer), 361–387.

[ref8] Aon Hewitt. (2015). Aon Hewitt’s Model of Employee Engagement. Available online at https://www.aon.com/attachments/human-capital-consulting/aon-hewitt-model-of-employee-engagement.pdf (Accessed September 28, 2026).

[ref9] Bailey C. Madden A. Alfes K. Fletcher L. (2017). The meaning, antecedents and outcomes of employee engagement: a narrative synthesis. Int. J. Manag. Rev. 19, 31–53. doi: 10.1111/ijmr.12077

[ref10] Bakker A. B. Demerouti E. Sanz-Vergel A. (2023). Job demands–resources theory: ten years later. Annu. Rev. Organ. Psychol. Organ. Behav. 10, 25–53. doi: 10.1146/annurev-orgpsych-120920-053933

[ref11] Bartunek J. M. Rynes S. L. (2010). The construction and contributions of "implications for practice": what’s in them and what might they offer? Acad. Manag. Learn. Educ. 9, 100–117. doi: 10.5465/amle.9.1.zqr100

[ref12] Blom M. Alvesson M. (2015). All-inclusive and all good: the hegemonic ambiguity of leadership. Scand. J. Manag. 31, 480–492. doi: 10.1016/j.scaman.2015.08.001

[ref13] Bourdieu P. (1984). Distinction: A Social Critique of the Judgement of Taste. Cambridge, MA: Harvard University Press.

[ref14] Bourdieu P. (1986). “The forms of capital,” in Handbook of Theory and Research for the Sociology of Education, ed. Richardson J. (New York: Greenwood Press), 241–258.

[ref15] Bourdieu P. (1989). Social space and symbolic power. Sociol Theory 7, 14–25. doi: 10.2307/202060

[ref16] Bourdieu P. (1990). The Logic of Practice. Stanford, CA: Stanford University Press.

[ref17] Bourdieu P. (2005). The Social Structures of the Economy. Cambridge: Polity Press.

[ref18] Brodie R. J. Hollebeek L. D. Jurić B. Ilić A. (2011). Customer engagement: conceptual domain, fundamental propositions, and implications for research. J. Serv. Res. 14, 252–271. doi: 10.1177/1094670511411703

[ref19] Christian M. S. Garza A. S. Slaughter J. E. (2011). Work engagement: a quantitative review and test of its relations with task and contextual performance. Pers. Psychol. 64, 89–136. doi: 10.1111/j.1744-6570.2010.01203.x

[ref20] Crawford E. R. LePine J. A. Rich B. L. (2010). Linking job demands and resources to employee engagement and burnout: a theoretical extension and meta-analytic test. J. Appl. Psychol. 95, 834–848. doi: 10.1037/a0019364, 20836586

[ref21] Csikszentmihalyi M. (1975). Beyond Boredom and Anxiety. San Francisco: Jossey-Bass.

[ref22] Csikszentmihalyi M. (1990). Flow: The Psychology of Optimal Experience. New York: Harper & Row.

[ref23] Cunliffe A. (2014). A Very Short, Fairly Interesting and Reasonably Cheap Book about Management. 2nd Edn London: Sage.

[ref24] Daft R. L. (2021). Organization Theory and Design. 13th Edn Boston, MA: Cengage.

[ref25] Demerouti E. Bakker A. B. Nachreiner F. Schaufeli W. B. (2001). The job demands-resources model of burnout. J. Appl. Psychol. 86, 499–512. doi: 10.1037/0021-9010.86.3.499, 11419809

[ref26] Emirbayer M. Johnson V. (2008). Bourdieu and organizational analysis. Theory Soc. 37, 1–44. doi: 10.1007/s11186-007-9052-y

[ref27] Fredricks J. A. Blumenfeld P. C. Paris A. H. (2004). School engagement: potential of the concept, state of the evidence. Rev. Educ. Res. 74, 59–109. doi: 10.3102/00346543074001059

[ref28] George J. M. (2011). The wider context, costs, and benefits of work engagement. Eur. J. Work Organ. Psychol. 20, 53–59. doi: 10.1080/1359432X.2010.509924

[ref29] Grey C. (2017). A Very Short, Fairly Interesting and Reasonably Cheap Book about Studying Organizations. 4th Edn London: Sage.

[ref30] Harter J. K. Schmidt F. L. Hayes T. L. (2002). Business-unit-level relationship between employee satisfaction, employee engagement, and business outcomes: a meta-analysis. J. Appl. Psychol. 87, 268–279. doi: 10.1037/0021-9010.87.2.268, 12002955

[ref31] Hobfoll S. E. Halbesleben J. Neveu J.-P. Westman M. (2018). Conservation of resources in the organizational context: the reality of resources and their consequences. Annu. Rev. Organ. Psychol. Organ. Behav. 5, 103–128. doi: 10.1146/annurev-orgpsych-032117-104640

[ref32] Iles P. Yolles M. (2003). “Complexity, HRD and organisation development: towards a viable systems approach to learning, development and change,” in HRD in A Complex World, ed. Lee M. (London: Routledge), 25–41.

[ref33] Jayanti E. B. (2011). Through a different lens: a survey of linear epistemological assumptions underlying HRD models. Hum. Resour. Dev. Rev. 10, 101–114. doi: 10.1177/1534484310386753

[ref34] Kahn W. A. (1990). Psychological conditions of personal engagement and disengagement at work. Acad. Manag. J. 33, 692–724. doi: 10.2307/256287

[ref35] Kahu E. R. (2013). Framing student engagement in higher education. Stud. High. Educ. 38, 758–773. doi: 10.1080/03075079.2011.598505

[ref36] Kantola A. Seeck H. (2011). Dissemination of management into politics: Michael Porter and the political uses of management consulting. Manag. Learn. 42, 25–47. doi: 10.1177/1350507610382489

[ref37] Keenoy T. (2014). “Engagement: a murmuration of objects?” in Employee Engagement in Theory and Practice, eds. Truss C. Delbridge R. Alfes K. Shantz A. Soane E. (London: Routledge), 197–220.

[ref38] Kerlinger F. N. Lee H. B. (2000). Foundations of Behavioral Research. 4th Edn Fort Worth: Harcourt College Publishers.

[ref39] Kessler S. R. Gutworth M. B. (2023). The forgotten working class: a call to action based upon a repeated cross-sectional examination of the relationships among social class, financial satisfaction, and exhaustion. Group Organ. Manag. 48, 1306–1338. doi: 10.1177/10596011221099797

[ref40] Klassen R. M. Yerdelen S. Durksen T. L. (2013). Measuring teacher engagement: development of the engaged teachers scale (ETS). Front. Learn. Res. 1, 33–52. doi: 10.14786/flr.v1i2.44

[ref41] Learmonth M. Morrell K. (2019). Critical Perspectives on Leadership: The Language of Corporate Power. New York: Routledge.

[ref42] Lee Y. Shin H. Y. Park J. Kim W. Cho D. (2017). An integrative literature review on employee engagement in the field of human resource development: exploring where we are and where we should go. Asia Pac. Educ. Rev. 18, 541–557. doi: 10.1007/s12564-017-9508-3

[ref43] Leroy S. (2009). Why is it so hard to do my work? The challenge of attention residue when switching between work tasks. Organ. Behav. Hum. Decis. Process. 109, 168–181. doi: 10.1016/j.obhdp.2009.04.002

[ref44] Macey W. H. Schneider B. (2008). The meaning of employee engagement. Ind. Organ. Psychol. 1, 3–30. doi: 10.1111/j.1754-9434.2007.0002.x

[ref45] May D. R. Gilson R. L. Harter L. M. (2004). The psychological conditions of meaningfulness, safety and availability and the engagement of the human spirit at work. J. Occup. Organ. Psychol. 77, 11–37. doi: 10.1348/096317904322915892

[ref46] Newman D. A. Harrison D. A. (2008). Been there, bottled that: are state and behavioral work engagement new and useful construct "wines"? Ind. Organ. Psychol. 1, 31–35. doi: 10.1111/j.1754-9434.2007.00003.x

[ref47] Newman D. A. Joseph D. L. Sparkman T. E. Carpenter N. C. (2011). Invited reaction: the work cognition inventory: initial evidence of construct validity. Hum. Resour. Dev. Q. 22, 37–47. doi: 10.1002/hrdq.20065

[ref48] Newport C. (2016). Deep Work: Rules for Focused Success in a Distracted World. New York: Grand Central Publishing.

[ref49] Rich B. L. LePine J. A. Crawford E. R. (2010). Job engagement: antecedents and effects on job performance. Acad. Manag. J. 53, 617–635. doi: 10.5465/amj.2010.51468988

[ref50] Rigg C. Stewart J. Trehan K. (eds.) (2007). Critical Human Resource Development: Beyond Orthodoxy. Harlow: Pearson Education.

[ref51] Russell B. (1979). A History of Western Philosophy. London: George Allen & Unwin.

[ref52] Ryan R. M. Deci E. L. (2001). On happiness and human potentials: a review of research on hedonic and eudaimonic well-being. Annu. Rev. Psychol. 52, 141–166. doi: 10.1146/annurev.psych.52.1.141, 11148302

[ref53] Saks A. M. (2006). Antecedents and consequences of employee engagement. J. Manag. Psychol. 21, 600–619. doi: 10.1108/02683940610690169

[ref54] Saks A. M. Gruman J. A. (2014). What do we really know about employee engagement? Hum. Resour. Dev. Q. 25, 155–182. doi: 10.1002/hrdq.21187

[ref55] Sambrook S. (2021). The life-cycle of engagement: towards a divergent critical HR/pluralist conceptualization. Hum. Resour. Dev. Int. 24, 468–489. doi: 10.1080/13678868.2021.1961508

[ref56] Sartori G. (1970). Concept misformation in comparative politics. Am. Polit. Sci. Rev. 64, 1033–1053. doi: 10.2307/1958356

[ref57] Sartre J.-P. (2007). Existentialism is a Humanism. New Haven, CT: Yale University Press (Original work published 1946).

[ref58] Sartre J.-P. (2018). Being and Nothingness: An Essay in Phenomenological Ontology. New York: Routledge. (Original work published 1943).

[ref59] Schaufeli W. B. Bakker A. B. Salanova M. (2006). The measurement of work engagement with a short questionnaire: a cross-national study. Educ. Psychol. Meas. 66, 701–716. doi: 10.1177/0013164405282471

[ref60] Schaufeli W. B. Salanova M. González-Romá V. Bakker A. B. (2002). The measurement of engagement and burnout: a two sample confirmatory factor analytic approach. J. Happiness Stud. 3, 71–92. doi: 10.1023/A:1015630930326

[ref61] Schimmelpfennig R. Elbæk C. Mitkidis P. Singh A. Roberson Q. (2025). The "WEIRDEST" organizations in the world? Assessing the lack of sample diversity in organizational research. J. Manag. 51, 2460–2487. doi: 10.1177/01492063241305577, 40470034 PMC12133052

[ref62] Shaffer J. A. DeGeest D. Li A. (2016). Tackling the problem of construct proliferation: a guide to assessing the discriminant validity of conceptually related constructs. Organ. Res. Methods 19, 80–110. doi: 10.1177/1094428115598239

[ref63] Shirom A. (2003). “Feeling vigorous at work? The construct of vigor and the study of positive affect in organizations,” in Emotional and Physiological Processes and Positive Intervention Strategies (Research in Occupational Stress and Well-Being), eds. Perrewé P. L. Ganster D. C., vol. 3 (Bingley: Emerald), 135–164.

[ref64] Shuck B. Adelson J. L. Reio T. G. Jr. (2017a). The employee engagement scale: initial evidence for construct validity and implications for theory and practice. Hum. Resour. Manag. 56, 953–977. doi: 10.1002/hrm.21811

[ref65] Shuck B. Collins J. C. Rocco T. S. Diaz R. (2016). Deconstructing the privilege and power of employee engagement: issues of inequality for management and human resource development. Hum. Resour. Dev. Rev. 15, 208–229. doi: 10.1177/1534484316643904

[ref66] Shuck B. Ghosh R. Zigarmi D. Nimon K. (2013). The jingle jangle of employee engagement: further exploration of the emerging construct and implications for workplace learning and performance. Hum. Resour. Dev. Rev. 12, 11–35. doi: 10.1177/1534484312463921

[ref67] Shuck B. Osam K. Zigarmi D. Nimon K. (2017b). Definitional and conceptual muddling: identifying the positionality of employee engagement and defining the construct. Hum. Resour. Dev. Rev. 16, 263–293. doi: 10.1177/1534484317720622

[ref68] Shuck B. Wollard K. (2010). Employee engagement and HRD: a seminal review of the foundations. Hum. Resour. Dev. Rev. 9, 89–110. doi: 10.1177/1534484309353560

[ref69] Siehl C. Martin J. (1984). “The role of symbolic management: how can managers effectively transmit organizational culture?” in Leaders and Managers: International Perspectives on Managerial Behavior and Leadership, eds. Hunt J. G. Hosking D.-M. Schriesheim C. A. Stewart R. (New York: Pergamon Press), 227–239.

[ref70] Soane E. Truss C. Alfes K. Shantz A. Rees C. Gatenby M. (2012). Development and application of a new measure of employee engagement: the ISA engagement scale. Hum. Resour. Dev. Int. 15, 529–547. doi: 10.1080/13678868.2012.726542

[ref71] Spoelstra S. ten Bos R. (2011) Leadership Continental Philosophy and Business Ethics Painter-Morland M. ten Bos R. Cambridge: Cambridge University Press 181–198

[ref72] Suddaby R. (2010). Editor’s comments: construct clarity in theories of management and organization. Acad. Manag. Rev. 35, 346–357. doi: 10.5465/amr.35.3.zok346

[ref73] Truss C. Shantz A. Soane E. Alfes K. Delbridge R. (2013). Employee engagement, organisational performance and individual well-being: exploring the evidence, developing the theory. Int. J. Hum. Resour. Manag. 24, 2657–2669. doi: 10.1080/09585192.2013.798921

[ref74] van Knippenberg D. Sitkin S. B. (2013). A critical assessment of charismatic-transformational leadership research: back to the drawing board? Acad. Manag. Ann. 7, 1–60. doi: 10.1080/19416520.2013.759433

[ref75] Virág C. (2019). “Moral psychology and cultivating the self,” in Zhu Xi: Selected Writings, ed. Ivanhoe P. J. (New York: Oxford University Press), 35–55.

[ref76] Wittenberg H. Eweje G. Taskin N. Forsyth D. (2024). Different perspectives on engagement, where to from here? A systematic literature review. Int. J. Manag. Rev. 26, 410–434. doi: 10.1111/ijmr.12361

[ref77] Xanthopoulou D. Bakker A. B. Demerouti E. Schaufeli W. B. (2009). Work engagement and financial returns: a diary study on the role of job and personal resources. J. Occup. Organ. Psychol. 82, 183–200. doi: 10.1348/096317908X285633

[ref78] Yang C. O’Leary S. Tregidga H. (2021). Social impact in accounting: is it at risk of becoming a hembig concept and does this matter? Qual. Res. Account. Manag. 18, 313–331. doi: 10.1108/QRAM-05-2021-0093

[ref79] Yawson R. M. (2013). Systems theory and thinking as a foundational theory in human resource development – a myth or reality? Hum. Resour. Dev. Rev. 12, 53–85. doi: 10.1177/1534484312461634

